# Improving Healing: The Putative Effects of Low-Level Laser Therapy for Ulcer in Parkinson's Disease

**DOI:** 10.7759/cureus.56756

**Published:** 2024-03-23

**Authors:** Anam R Sasun, Pratik Phansopkar, Moh'd Irshad Qureshi

**Affiliations:** 1 Department of Neuro-Physiotherapy, Ravi Nair Physiotherapy College, Datta Meghe Institute of Higher Education and Research (DMIHER), Wardha, IND; 2 Department of Musculoskeletal Physiotherapy, Ravi Nair Physiotherapy College, Datta Meghe Institute of Higher Education and Research (DMIHER), Wardha, IND

**Keywords:** physiotherapy, orif, fracture, proximal tibia fibula, masked shaped face, resting tremors, parkinson

## Abstract

The progressive nature of Parkinson's disease and its associated motor and non-motor symptoms can lead to various complications when patients experience immobilization, exacerbating existing motor impairments and potentially giving rise to secondary health issues. The variability, progression, and management of tremors in PD can be challenging. Due to low bone mass density, patients with Parkinson's disease are susceptible to vitamin D deficiency. The lack of movement can worsen muscle rigidity and stiffness, leading to contractures and a decreased range of motion in joints. Additionally, immobility may contribute to cardiovascular deconditioning, orthostatic hypotension, and an increased risk of pressure ulcers due to prolonged pressure on specific areas of the body. In this case report, we hereby report a case of Parkinson's disease further complicated by sinus discharge from the ulcer. This case report describes the putative effects of low-level laser therapy on discharging sinus from the wound secondary to a diabetic ulcer in idiopathic Parkinson's disease. Achieving an ideal level of functional independence and preventing problems associated with extended immobility are essential goals of structured physical therapy postoperative care. This may assist the patient in returning to their pre-injury position more quickly. Our patient underwent several interventions for wound healing, including proprioception training, tremor management, improving dynamic trunk balance, and pain control measures. Clinical outcome measures like the Barthel Index, lower extremity functional scale, and Visual-Analog Scale were used to assess the progress of the patient. Managing these interconnected conditions requires a multi-disciplinary approach.

## Introduction

Parkinson's disease (PD) is a neurodegenerative disorder that progresses and is characterized by tremors, bradykinesia, rigidity, and postural instability. A broader and more diverse range of motor and non-motor symptoms is emerging [1]. Resting tremors are the most common motor symptom, affecting 75% of diseased patients. It occurs most frequently in the distal extremities and has a frequency of 3 to 6 Hz [2]. The intricacies involved in managing and progressing through tremors in PD, particularly in the tremor-dominant subtype, underscore the complexity of treatment. Non-motor symptoms of Parkinson's disease include digestive problems, sleep problems, and behavioral and cognitive changes that can manifest years before motor symptoms. It is a disease with shifting complexities [3]. Patients with Parkinson's disease are more prone to falling and have a higher risk of fractures because of their fragile nature. Falls cause significant morbidity in Parkinson's disease patients, limiting their daily activities [4].

Low-level laser therapy works wonders for wound healing. The laser utilized exhibited an energy density of 10 J/cm^2^ and operated at a wavelength of 685 nm. Analysis of ulcer size reduction alongside complete healing revealed that low-level laser therapy (LLLT) could accelerate the resolution of persistent foot ulcers., ultimately decreasing the overall duration required for complete healing [5]. The anti-inflammatory effects of laser therapy can be explained by the inhibition of prostaglandins, interleukins, and cytokines in cell and animal models [6]. Physical therapy sessions have been shown to slow disease progression and improve overall function. Aerobic and eccentric exercises, as well as hand movements and cycling, have been shown to help with tremors [7]. Fractures of the tibia and fibula in the elderly are uncommon, but when they do occur, they can be extremely painful. Based on Medicare population data in the United States [8]. Tibia and fibula shaft fractures can cause serious catastrophic consequences like soft tissue infection, malunion, and necrosis. We hereby report a case of idiopathic Parkinson's disease further complicated by a compound grade II 1/3rd right tibia and fibula fracture managed with ORIF (open-reduction and internal fixation) and osteosynthesis plating that was further complicated by discharging sinus from the wound secondary to a diabetic ulcer. Here, we discuss the physiotherapy management of the stated instance and emphasize how important physiotherapy is to the patient's return to his normal life. Thus enhancing life quality in the process.

## Case presentation

Patient information

After undergoing a surgical procedure on his right lower extremity, a 65-year-old retired male police officer was admitted to the orthopedic ward for males. with a significant medical history of idiopathic Parkinson's disease and diabetes diagnosed five years ago. He reported pain and swelling at the surgical site, along with discharge from the surgical site. The pain was localized to the medial and lateral aspects of the right leg, which were characterized by a dull, aching nature that aggravated during transfers and movement. Additionally, the patient complained of involuntary tremors in the right arm, which progressively affected the right hand, with distal arm tremors at the elbow joint occurring at a rate of approximately five to six per second. The patient was seemingly well two months ago when he suffered a tripping injury on the stairs, resulting in an injury to his right lower limb. Following immediate attention at a nearby clinic, radiographic examinations revealed a compound Grade II 1/3rd right tibia and fibula fracture. Subsequently, the patient underwent ORIF (open-reduction and internal fixation ) with an osteosynthesis plate. The patient was released from the hospital, but he soon developed sinus discharge from the operating extremity. The pain associated with the ulcer was described as throbbing. Consequently, the patient was referred to another center, where the implant was removed and a sinus tract excision was performed. A 50-mm diabetic ulcer inside the cast was discovered. Fracture reduction was achieved through traction and manipulation, confirmed under C-arm, and advanced to distal fragment, and distal locking was done with 32 and 30-mm locking bolts. Following this, the patient was referred to physiotherapy for post-operative management and to address the symptoms of Parkinson's disease.

Clinical evaluation

**Figure 1:**
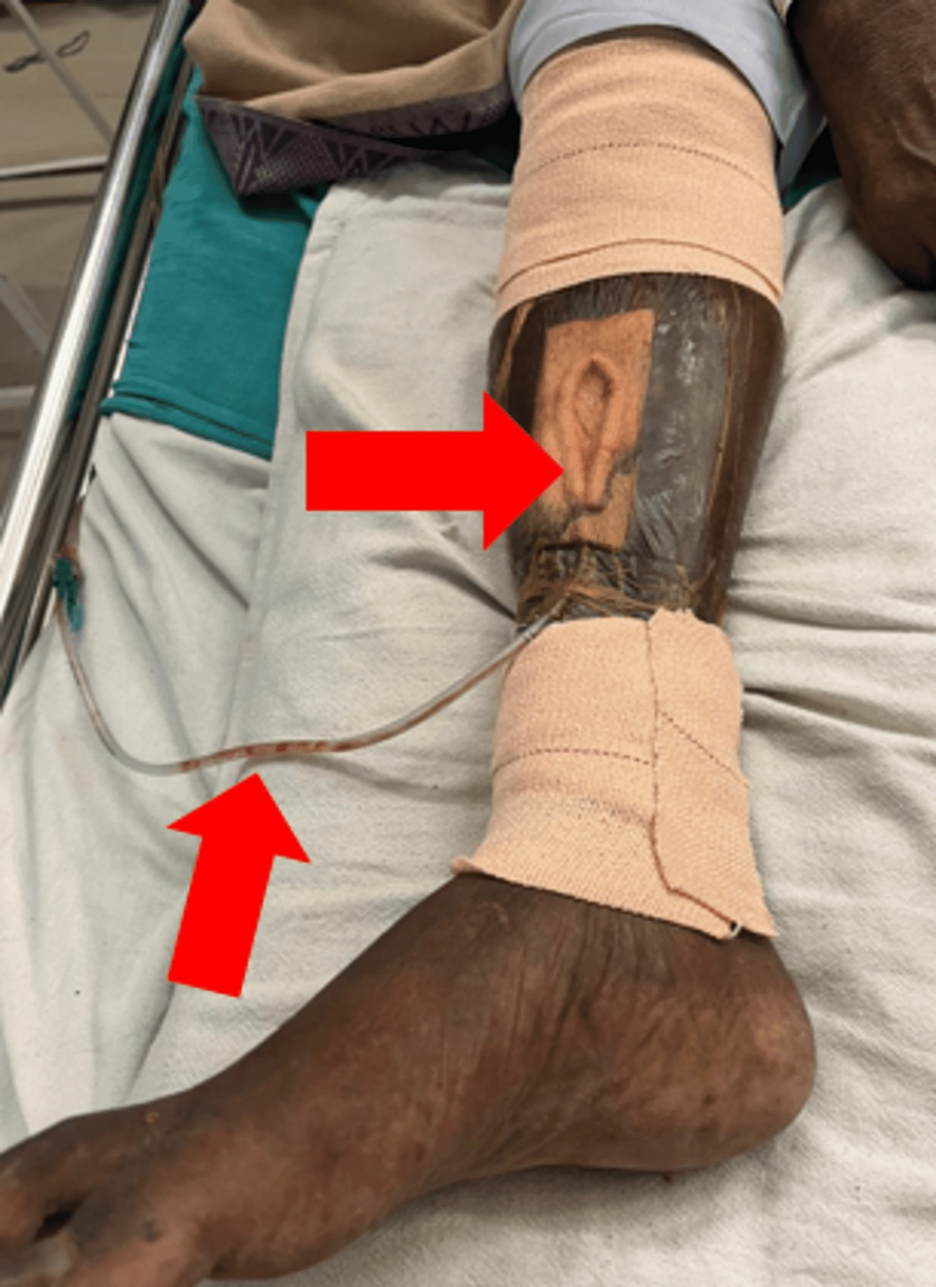
Ulcer over antero-medial aspect of the right leg and application of VAC VAC: Vacuum-Assisted Closure

Upon examination, the patient was positioned in a high-sitting posture with the operated extremity on a pillow, with the hip and knees flexed and the hip in external rotation. Notably, a yellowish discoloration was evident on the anterior aspect of the leg (Figure 1), and a vacuum-assisted closure was present to aid in wound healing. The patient also exhibited resting tremors and a masked-shaped face. Grade I swelling is present over the right foot, and a coarse, high-amplitude tremor, likely of moderate frequency (around five to six cycles per second), persists in the distal arm at the elbow joint, extending to the proximal arm when elevated. Palpation reveals Grade II tenderness at the suture site. Additionally, spasms are noted in the quadriceps muscle. During the examination, swelling is assessed using the Figure Eight method, resulting in a measurement of 33.5 cm per inch of tape. Similarly, the measurement of swelling in the left foot yields a value of 33 cm per inch of tape. Notably, it also exhibited a substantial amount of hyperkeratosis, swollen edges with exudation, and an unpleasant odor. Also, it confirmed the presence of Grade I swelling on the right leg. On examination, a range of motion assessment was done, which revealed that the strength grading for the right-sided quadriceps femoris and hamstring was weak and painful on resisted isometric muscle testing. On sensory examination, all superficial, deep, and combined cortical reflexes were intact. Girth measurements of the right and left thighs yielded atrophy of the quadriceps muscle.

Physiotherapy interventions

The regaining of mobility and functional activities of the patient depends on physiotherapy intervention. The designed protocol was planned in agreement with patient’s family. Tables 1, 2 describe broader aspects of physiotherapy interventions in managing symptoms of Parkinson and the complications of the fracture. Conventional therapy along-with low-level laser therapy was followed, and the use of laser therapy showed positive results on the wound bed.

**Table 1:** Physiotherapy rehabilitation for week 1 to week 2 W: Watt/cm^2^; Reps: repetitions

Sr No.	Goals	Interventions
1.	Prevention of further post-operative complications like chest infections and pressure sores.	Maintaining proper body alignment and positioning ensures cushioning of the prominent sites for pressure sores. Ankle toe movements (25 reps× 2 sets twice a day. Ensure that the patient's body is properly aligned when lying in bed or sitting in a chair. This includes keeping the head, shoulders, and hips in alignment to avoid unnecessary pressure on specific. Teaching effective huffing-coughing techniques.
2.	Educate the patient	Counseling about exercise regimens and the importance of adherence to them. Education about the further complications of diabetic ulcers, and Parkinson’s disease.
3.	To promote the healing of ulcer	Low-level Laser therapy for 15 minutes, 10 W power. Dosage 9 joules/cm^2^
4.	Reduce pain and inflammation at the fracture site	Crepe bandage application in the figure of “eight method” distal to proximal. Cryotherapy for 10 minutes, four to five times daily.
5.	To enhance sensory integration	Various stimulations such as light touch, deep pressure, tactile kinesthesia, and visual were used (10 repetitions-two sets each).
6.	Improve muscle strength	Isometric contraction of Quadriceps, hamstrings, and glutei (10 rapid contractions-two sets) withhold time being 5 seconds. Passive straight leg raise exercises (10 repetitions of two sets). Unilateral pelvic bridging (weight bearing on the left lower limb) 10 repetitions of 2 set.
7.	Improve resting tremors [9].	Limb eccentric training (33-45 min/session for six weeks, three days/week) Dual-task training
8.	Improve functions of facial Muscles	Facial muscle massage and facial muscle training

**Table 2:** Physiotherapy Rehabilitation for Week 3 to Week 4 W: Watt/cm^2^; Reps:  Repetition.

Sr No.	Goals	Interventions
1.	To improve muscle strength	Improve contraction of quadriceps, hamstrings, and glutei (10 rapid contractions-two sets) withhold time being 5 seconds. Strengthening exercises for unaffected left lower limbs and bilateral upper limbs. Strengthening for Vastus medialis oblique strengthening exercise 1 sets of 10 repetitions. Straight leg raise exercise. Dynamic quadriceps using theraband and weight cuff
2.	To Improve muscle strength	Deep breathing exercises, Spirometry (10 reps, two set)
3.	To initiate weight bearing on the affected leg and ambulation.	Pre-weight bearing exercises
		Partial weight-bearing toe-touch weight- bearing exercises. Later, ambulation with walking aids
4.	To promote ulcer healing	Laser therapy for eight minutes, 7 W power. Dosage 3.186 joules/cm^2^
5.	Improve Resting Tremors	Dual-task training Functional Electrical stimulator for 40 minutes. Eccentric limb exercises (33-45 min/session)
6.	Improve independence of the patient and promote gait training	Application of faradic stimulation to the dorsiflexor group of muscles
7.	Strengthening of pelvis and hip musculature	Pelvic Proprioceptive neuromuscular facilitation (stabilizing reversal) 10 reps into two sets
8.	To improve Proprioception	Proprioception training helps to improve stability, balance, and coordination Balance exercises: Exercises like Static-sitting with eyes closed sitting with neck and trunk rotation
		Sitting with controlled upper limb movement followed by resisted limb movement were commenced
		Static standing with feet apart –together were done, static balance with feet together and, eyes closed-open were done
		Each exercise was done 10 repetitions x three sets with rest intervals
		Coordination exercises: Frenkel's exercises in lying, and sitting were commenced. Exercises in lying include, leg raising to place the heel on a marked point, heel slides to a position as indicated by us
		Exercises in Sitting include: Stretching of one leg, to slide to a position as indicated by the therapist, change to standing, and then sit again. Each exercise was done with 15 repetitions × three sets with rest intervals
		Stretching of one leg, to slide to a position as indicated by therapist, change to standing and then sit again. Each exercise was done 15 repetition × three sets with rest intervals. Joint position sense exercises
9.	Gait Training	Once the lower extremity has gained sufficient strength, gait training will be initiated. Partial weight bearing was started, and further progression was made with full-weight bearing using a walker after eight weeks of surgery
		Gait re-training was done to refine the components of gait and further improve gait mechanics

The assesment of clinical outcome measures were done at Day 1 and Day last of physiotherapy rehabilitation (Table 3).

 

**Table 3:** Outcome Measures at Day 1 and the last day of rehabilitation (six weeks) ROM: Range of motion

Outcome Measures	Pre intervention	Post intervention
Visual Analogue Scale	7/10	3/10
Rom hip flexion range	30	100
Right knee flexion	30	80
Quadriceps strength (Oxford Scale)	3/5	4/5
Hamstring strength (Oxford Scale)	3/5	4/5
Lower extremity functional scale	20.62 % /100	58.3%/100
Berg balance scale	20/56 (high fall risk )	41/56 (low risk of fall)
Barthel index	30	60

## Discussion

According to Madadi et al. (2011), tibial fractures are prone to complications due to the lack of a soft tissue envelope around the bone [10]. Donohoe et al., in their study, concluded that 901 million people over the age of 60 worldwide account for 12% of the global population. This increases the risk of osteoporosis and further falls, which cause fractures, which further increase the risk of mortality and morbidity [11]. Bed rest and limb immobilization accelerate the physiological alterations linked with typical aging, including muscle mass and strength decline and impaired blood glucose regulation, even when energy intake is adjusted to sustain caloric equilibrium. Elderly individuals exhibit diminished capacity to recuperate from muscle disuse compared to their younger counterparts [12].

According to Daf et al., by combining physiotherapy and surgical alternatives, ankle-foot orthosis (AFO), or functional electrical stimulation (FES) may be used to deliver interventions that are precisely tailored to meet the individualized needs of their patients [13]. Eccentric-based resistance exercises, in particular, are beneficial in the treatment of tremors [14]. Functional electrical stimulation is the most effective at tremor reduction. It causes muscle contractions to modulate its inherent ability to suppress tremors [15]. Pahwa et al. (2019) conducted a study to determine the effectiveness of functional electrical stimulation. After 40 minutes of stimulation, patients with essential tremors had higher clinical scale scores on the Essential Tremor Rating Scale than the sham group. Although the outcomes appear promising, differences were only discovered in one item, and no reliable kinematics measures were offered [16].

Exercise plans that are designed effectively and carefully worked out can play an essential function in the continuum of care for patients who have experienced a fragility fracture by maximizing functional recovery and reducing the risk of falls and other accidents [14]. According to Pereira et al. (2020), the patient's healing process and tissue repair were effectively aided by treatment with an unfocused high-power laser at a dose of 3.18 J/cm2 applied once a week. There are benefits to using unfocused high-power lasers to treat poorly healing wounds, including shorter application times and painless sessions [15]. Studies in the scientific literature indicate that using low-level laser therapy to treat wounds speeds up the healing process by influencing the inflammatory and proliferative phases of the wound healing process. It also promotes a more orderly and harmonious healing process, which improves the scar tissue's aesthetic outcomes [16].

## Conclusions

This study emphasizes the rehabilitation of a patient with a diabetic ulcer secondary to fracture cast application in idiopathic Parkinson's disease. A cascade of events occurs following the immobilization of the fractured extremity. People affected by idiopathic Parkinson's disease are at higher risk of falls, which secondarily cause fractures of the extremities. Diabetic ulcers secondary to fracture cast application are a complication of immobilization in the elderly. Common underlying causes are poor glycemic control, foot deformities, underlying neuropathies, and peripheral vascular diseases. The most feared complication of such a complication is gangrene and amputation of the extremity. This causes permanent disability and a reduced quality of life. Treatment of diabetic wounds requires a multi-disciplinary approach. The patient demonstrated remarkable improvement in ulcer healing, tremor reduction, and increased muscle strength and proprioception.
